# Supplementary material for: HIVprotI: an integrated web based platform for prediction and design of HIV proteins inhibitors
Source: J Cheminform. 2018 Mar 9;10:12. doi: 10.1186/s13321-018-0266-y (PMC5845081; doi:10.1186/s13321-018-0266-y)
Supplement: Supplementary file 2 — Additional file 2. Source code of HIVProtI web server. [file 13321_2018_266_MOESM2_ESM.zip › HIVprotI_Source-code/hivprotI/weka-3-6-6/doc/weka/gui/beans/TrainingSetProducer.html]

TrainingSetProducer


---


|  |  |  |
| --- | --- | --- |
| **Overview** \| **Package** \| **Class** \| **Tree** \| **Deprecated** \| **Index** \| **Help** \| **Weka's home** | |  |
| **PREV CLASS**   **NEXT CLASS** | **FRAMES**    **NO FRAMES**     **All Classes** |  |
| SUMMARY: NESTED \| FIELD \| CONSTR \| METHOD | DETAIL: FIELD \| CONSTR \| METHOD |  |


---


## weka.gui.beans Interface TrainingSetProducer

**All Known Implementing Classes:**: AbstractTrainAndTestSetProducer, AbstractTrainingSetProducer, ClassAssigner, CrossValidationFoldMaker, Filter, PredictionAppender, TrainingSetMaker, TrainTestSplitMaker

---

``` public interface TrainingSetProducer ```

Interface to something that can produce a training set

**Version:**
:   $Revision: 1.3 $

**Author:**
:   Mark Hall

---

| **Method Summary** | |
| --- | --- |
| `void` | `addTrainingSetListener(TrainingSetListener tsl)`             Add a training set listener |
| `void` | `removeTrainingSetListener(TrainingSetListener tsl)`             Remove a training set listener |

**Method Detail**

### addTrainingSetListener

```
void addTrainingSetListener(TrainingSetListener tsl)
```

:   Add a training set listener

    :   **Parameters:**: `tsl` - a `TrainingSetListener` value

---


### removeTrainingSetListener

```
void removeTrainingSetListener(TrainingSetListener tsl)
```

:   Remove a training set listener

    :   **Parameters:**: `tsl` - a `TrainingSetListener` value


---


|  |  |  |
| --- | --- | --- |
| **Overview** \| **Package** \| **Class** \| **Tree** \| **Deprecated** \| **Index** \| **Help** \| **Weka's home** | |  |
| **PREV CLASS**   **NEXT CLASS** | **FRAMES**    **NO FRAMES**     **All Classes** |  |
| SUMMARY: NESTED \| FIELD \| CONSTR \| METHOD | DETAIL: FIELD \| CONSTR \| METHOD |  |


---
